# Culture Is Key: Engaging Culturally and Linguistically Diverse Populations in Breast Cancer Screening in High Income Contexts: A Scoping Review

**DOI:** 10.1002/cam4.70817

**Published:** 2025-03-28

**Authors:** Karla Jaques, Thomas Baker, Deepak Maharaj, Mohammed Fazli, Mandy Williams, Patrick Harris

**Affiliations:** ^1^ Centre for Health Equity, Training, Research and Evaluation (CHETRE), Part of the International Centre for Future Health Systems University of New South, South Western Sydney Local Health District Population Health Liverpool New South Wales Australia; ^2^ Breast Screen South Western Sydney Local Health District Population Health Liverpool New South Wales Australia; ^3^ South Western Sydney Local Health District Population Health Liverpool New South Wales Australia

**Keywords:** breast screening, CALD, cancer screening, culturally and linguistically diverse, healthcare disparities, mammogram, minority, vulnerable populations

## Abstract

**Background:**

Breast cancer is the most commonly diagnosed cancer in Australia and is the second highest cause of cancer mortality in Australian women. Screening in the form of mammography can significantly reduce mortality; however, research suggests that women from culturally and linguistically diverse (CALD) backgrounds are less likely to participate in mammography screening. While there is an established body of literature describing the lower engagement of CALD populations in screening and the associated challenges they face, less is known about evidence‐based interventions to improve engagement.

**Methods:**

A systematic scoping review was conducted to gain insights into best practice interventions to improve engagement of CALD populations in breast cancer screening. The search strategy followed the Preferred Reporting Items for Systematic Reviews and Meta‐Analyses extension for Scoping Reviews (PRISMA‐ScR) guidelines. PUBMED, EMBASE and CINHAL databases were searched for studies published between January 2012 and October 2023.

**Results:**

The search yielded 3249 studies; after removing duplicates, 2011 titles and abstracts were screened, and 121 papers underwent full text review. Forty‐one were included in the review. Key intervention types were identified, with combination or multi‐component studies being most effective at increasing mammography in CALD populations. Cultural appropriateness and tailoring are the most important considerations to be integrated into screening programs.

**Conclusion:**

CALD populations have lower engagement and experience many challenges in accessing screening services. This review found that the integration of cultural appropriateness and tailoring is critical in the successful delivery of breast screening services to CALD populations. Individual strategies are insufficient to engage this population in screening; multicomponent strategies are the most effective.

## Graphical abstract

Culturally and linguistically diverse populations have lower engagement and experience multiple challenges accessing breast screening services. Specific culturally appropriate, tailored, multi‐component strategies are necessary to improve engagement in breast screening services for these populations.

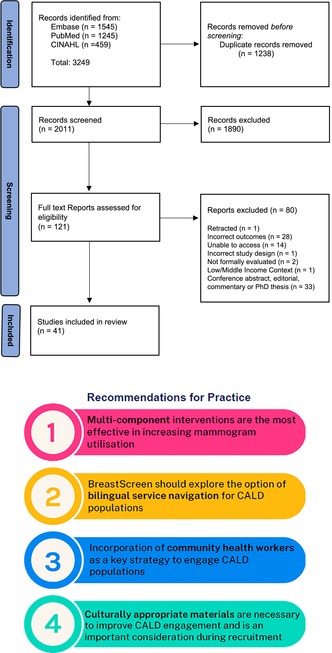

## 1 Introduction

Breast cancer is the most commonly diagnosed cancer globally and accounted for 685,000 cancer‐related deaths in 2020 [1]. Screening for cancer is an important preventative strategy as it allows for early detection, diagnosis and treatment, resulting in increased cancer survivorship and reduced mortality [2, 3]. Similarly, a systematic review [4] found that worldwide, women who use mammography breast screening programs can significantly reduce breast cancer mortality by 33%.

Despite the evidence linking mammography to reduced mortality, guidelines for who should receive mammograms vary from country to country. Within the United Kingdom and Australia, mammograms are free for residents invited to attend the appointment [5, 6]. However, other western countries like the United States of America and Canada have varying payment regimens associated with mammography screening, which is administered mostly through the primary care setting [7, 8].

In Australia, where this review was conducted, mammograms are free for citizens and permanent residents aged 50–74 through the national screening program *BreastScreen*, which provides biennial mammograms to this population [5]. Unfortunately, despite mammography availability, the program is yet to meet its target to screen 70% of eligible individuals [9].

Research suggests that women from a culturally and linguistically diverse (CALD) [1, 10] backgrounds are less likely to have awareness of or participate in cancer screening [9, 11, 12]. Data from the BreastScreen Australia Monitoring Report 2021 [9] indicates that women in Australia who speak a language other than English at home have a lower participation rate in breast screen services than their English‐speaking counterparts (45.5% vs. 56.2%).

An established body of literature demonstrates lower engagement in breast cancer screening from CALD populations and the associated challenges they face. Less is known about evidence‐based interventions to improve this engagement. This paper aims to explore the best practice principles to improve the engagement of CALD populations in breast cancer screening. The research questions were as follows:

1. Explore the approaches to engage CALD populations in preventive cancer screening
  ○ What types of interventions have been utilized?
  ○ Have these interventions been evaluated for their effectiveness and impact?

## 2 Materials and Methods

The systematic scoping review was conducted to gain insights into the main concepts, theories and knowledge gaps around CALD engagement in breast screening services; especially among cultural groups found within South Western Sydney Local Health District (SWSLHD) [13]. The search strategy followed a Preferred Reporting Items for Systematic Reviews and Meta‐Analyses extension for Scoping Reviews (PRISMA ScR) guidelines [13] and was developed in consultation with a research librarian as well as ongoing consultation with SWSLHD BreastScreen staff. Initial testing of the search terms was conducted and ‘model papers’ were identified and used to assist with testing and refinement of the search terms. Based upon initial testing, the search terms were expanded to specific language and cultural groups found within SWSLHD. This was done to better understand how interventions and services could be altered to create a more inclusive, patient‐centred and justice‐informed service framework. As per the Cochrane Rapid Review Guidelines [14] through consultation with SWSLHD BreastScreen, a PICO (Population, Intervention, Comparison, Outcomes) was developed (Table 1).

**TABLE 1:** PICO Framework.

Population or problem	CALD populations and effective engagement with BreastScreen
Intervention or exposure	Intervention aimed at improving engagement in mammography screening services
Comparison	N/A
Outcome	Service delivery Service usage Access

Peer reviewed articles were identified through an electronic search of studies published between January 2012 and October 2023 across three databases: PUBMED, EMBASE and CINHAL to cover multidisciplinary databases. A set of search terms (Table 2) used for each area was compiled. The database search results were imported into a single library in Covidence systematic review software (Veritas Health Information Australia), where duplicates were removed and remaining results underwent primary (title/abstract) and secondary (full text) screening.

**TABLE 2:** Search term groups were combined with the Boolean operator ‘AND’.

Search #1
“Culturally and linguistically diverse” OR “CALD” OR “non‐English speaking” OR “non‐english‐speaking” OR “ethnicity” OR “minority” OR “ethnic” OR “racialized” OR “migrant” OR “refugee” OR “BAME” OR “BME” OR “English as a second language” OR “ESL” or “Cross cultural” OR “Cross‐cultural” OR “Multicult*” OR “Vietnamese*” OR “Arabic*” OR “Chinese*” OR “Spanish*” OR “Italian*”
Search #2
“cancer screening” OR “Breast Screen*” OR “cancer prevention” OR “mammogram” OR “BreastScreen” OR “Breast cancer screening” OR “early detection of cancer”
Search #3
“access” OR “service usage” OR “service delivery” OR “awareness” OR “screening rat*” OR “Knowledge” OR “participat*” OR “Intention to screen” OR “Screening participation”
Search #4
Assess* OR Evaluat* OR monitor* OR Review OR Intervention* OR Investigat*

### 2.1 Inclusion and Exclusion Criteria

Articles were included in the review if they were as follows: (i) peer‐reviewed; (ii) evaluated or monitored an intervention policy or program targeting CALD engagement in cancer screening; (iii) reported an outcome that related to accessing screening services; (iv) mentioned ‘Breast’ in the title or abstract; (v) high income context [15]; (vi) published between 2012 and 2023, and (vii) in English. Articles were excluded if they were: (i) study protocols, commentary's, editorials or books/theses; (ii) only described ‘the problem’ no solutions/interventions described; (iii) did not include an evaluative component; (iv) did not report a screening related outcome; (v) interventions that are payment/removal of payment focused (A large number of interventions [primarily from the USA] utilised free or subsided screening as their primary intervention strategy. As this approach is not relevant to the Australian context [BreastScreen] these studies were excluded. Studies that used this approach in combination with other interventions were considered.); (vi) or did not contribute meaningfully to answering the research question, purpose or objectives. Grey literature was excluded as the review was focused on established, best practice literature on interventions to improve CALD engagement in breast cancer screening services.

### 2.2 Study Selection

Using the inclusion and exclusion criteria, titles and abstracts of retrieved articles were assessed by two independent reviewers, with the initial 10% of the sample being double screened, and conflict resolution took place to reduce the risk of selection bias. All full text articles were reviewed by two independent reviewers. Disagreements were resolved by discussion.

### 2.3 Data Extraction and Syntheses

Categorical data from each article (author, year, country, setting, target population and sample, approach and outcomes) were extracted. Each article included in the final synthesis underwent a narrative synthesis of the key findings of approaches to engage CALD populations in screening services and related outcomes.

## 3 Results

**FIGURE 1:**
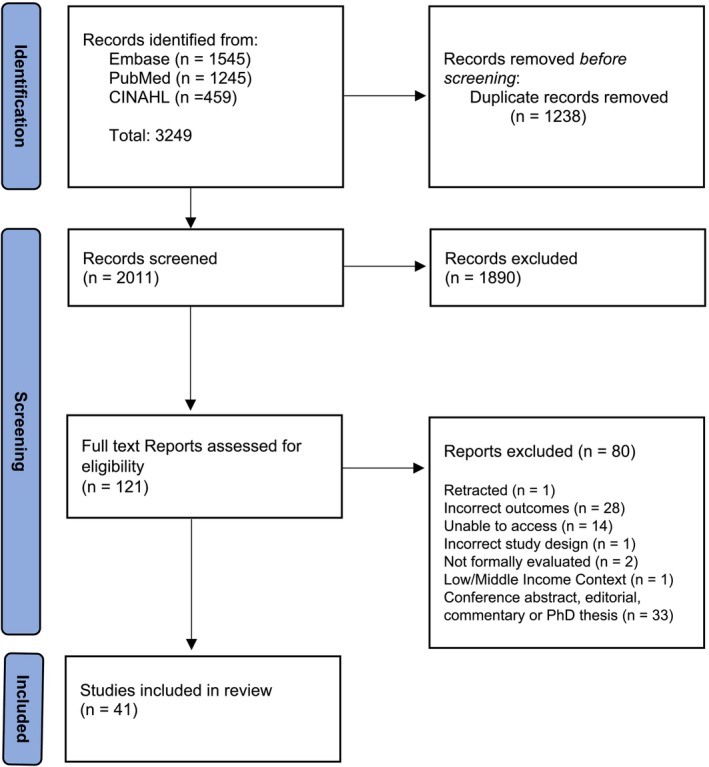
PRISMA Diagram of database search.

The database search identified 3249 potentially relevant articles. After duplicates were removed, 2011 titles and abstracts were screened. Of these, a total of 121 full text articles were screened, with 80 titles being excluded, leaving 41 articles for inclusion in this review (Figure 1).

The characteristics of the 41 studies that met the inclusion criteria are outlined in Appendix: Table A1. Most studies were from the USA (*n* = 35), followed by Australia (*n* = 3), UK (*n* = 1) Korea (*n* = 1) and multiple countries (*n* = 8). There were 28 intervention studies (5 randomised control trials and 23 quasi‐experimental) and 13 systematic review articles. Studies were primarily implemented in the community setting (*n* = 24), followed by a combination of settings (*n* = 9), primary care (*n* = 6) and national/state screening programmes (*n* = 2). Of the included studies, the majority were multi‐component interventions (*n* = 38); however, there was a small number of singular focus intervention studies (*n* = 3). Target populations varied, with most studies targeting women who were due or in need of a mammogram or, alternatively, women who had lapsed recommended follow‐up periods.

Importantly, the reviewed articles measured mammography outcomes in different ways. Around half of the included studies had multiple outcome measures (*n* = 20). Most studies referenced mammography through mammography completion (*n* = 37), while other studies referred to mammography utilisation as mammography bookings (*n* = 3). Other outcomes included intention to undergo mammography, cancer knowledge, behaviours, attitudes and beliefs, awareness of screening services, readiness to change, self‐breast examination, timely follow‐up with abnormal results and screening adherence.

We present the results that are organised below by intervention approach and other key findings from the literature.

### 3.1 Intervention Types

#### 3.1.1 Multi‐Component

Most of the intervention literature reviewed included multi‐component interventions defined as employing a combination of 2 or more interventions with the whole study or individual study arms, for example, patient navigation provided by a lay health worker in a faith‐based setting, where the navigation includes assistance booking appointments but also education around the availability of screening services (combining patient navigation, cultural appropriateness and education) versus an individual intervention of providing a mobile mammography service or reminder from a physician. Of the multicomponent study arms, a combination of three intervention types; education, patient navigation, and culturally appropriate and tailored interventions saw the greatest improvements in mammogram utilization. These are exemplified by the Molowku et al. study [16], the Gondek et al. study [17] and the Percac‐Lima et al. study (2012) [18] which saw improvements in mammogram utilization of 35%–93% when compared to control groups. Among review articles, the most common combination strategy employed was the use of culturally appropriate materials or the use of patient navigators, community health workers or Promotoras (lay Hispanic/Latino community health educator/s).

Culturally appropriate education sessions were seen to be effective in many of the included studies which used this model as part of their whole study or specific study arms (*n* = 8) [19, 20, 21, 22, 23, 24, 25, 26, 27]. Patient navigators, community health workers or Promotoras were seen to be effective when used in combination with other strategies within Hou et al. [20], Liu et al. [21], Luque et al. [23] and Racine et al. [24] review articles. Additionally, Okasako‐Schmucker et al. [28] and Roland et al. [26], focus on the use of community health workers and patient navigators specifically; each showing considerable improvement in mammogram utilisation within their reviewed studies.

### 3.2 Navigation

Navigation that is providing assistance such as translation services, helping patients schedule appointments or navigating health services. Navigation systems varied in scope, responsibilities, and level of aid provided to patients. For example, in the study described by Percac‐Lima et al. 2013 [29], patient navigators would follow up with patients who received reminder letters with either in‐person visits or a phone call, conversed with patients in their preferred language, educated patients on the need for a mammogram, assisted in booking mammogram appointments, reminded patients of their appointments, arranged transport, resolved insurance problems, and sometimes accompanied patients to their appointments. Conversely, other navigation systems simply assisted in booking appointments and sometimes conversed in the patient's preferred language [17]. A large (39 studies) systematic review [28] found that studies where community health workers were utilized in care coordination, case management or navigation saw the largest increase in cancer screening uptake.

Of the intervention studies, the most effective navigation intervention utilized a multifaceted, culturally tailored intervention using education and navigation delivered by community health workers to improve mammography rates [16]. Compared with controls, 97% of the intervention group received a mammogram within a 4‐month period of the intervention, compared with 4% of the control group. Multicomponent studies, as stated above, were common even with navigation and evidenced increases in mammography utilization [16, 17, 22, 24, 29, 30, 31, 32, 33, 34, 35, 36, 37].

A study that used patient navigation alone, when compared to education alone or navigation and education in combination, found that the most effective study arm was navigation (74.3%), followed by the combination (62.9%) then education alone (28.4%) [38]. Other studies that used the patient navigation approach alone also recorded a similar positive impact [18, 39].

Long term investment of resources was highlighted as an important component in the delivery of navigation interventions. A study exploring the long term outcomes of patient navigation for refugee women found that although at the end of the program screening was significantly higher than baseline, over time this decreased in refugee women while remaining stable in English speaking women over a 5 year period [40]. This was also highlighted in the review article [26] which found that while patient navigation is successful in improving screening in both primary care and community settings, particularly for vulnerable populations, supporting these in resource limited settings is difficult.

The majority of the patient navigation interventions incorporated culture or language in some way, often through the use of staff (community health workers, patient navigators, lay health workers) from the target communities, that were frequently bilingual/bicultural [16, 17, 18, 22, 24, 26, 28, 31, 32, 33, 34, 35, 36, 37, 38, 39, 40]. However, whether this is a defining feature of the effectiveness of navigation is less clear.

### 3.3 Education

Education referred to interventions that used group‐based learning, bilingual sessions, one‐on‐one education with or without take‐home self‐learning materials. Education was commonly used with culturally appropriate materials [23, 41, 42, 43, 44, 45] or other intervention strategies [12, 16, 17, 18, 19, 20, 21, 22, 24, 25, 27, 28, 29, 30, 31, 33, 34, 35, 36, 38, 39, 45, 46, 47, 48]. Of these education interventions, the content, vehicle for delivery, and audiences varied considerably. Most papers utilized group‐based learning to improve breast cancer and mammography knowledge (*n* = 20) [12, 17, 20, 21, 22, 23, 24, 25, 28, 29, 33, 34, 35, 38, 42, 43, 45, 47, 48, 49]. Several papers used bilingual sessions to convey information (*n* = 17) [16, 17, 18, 22, 24, 29, 30, 31, 34, 35, 36, 38, 39, 41, 46, 47, 49], followed by one‐on‐one education (*n* = 15) [18, 20, 21, 22, 23, 24, 28, 29, 30, 34, 39, 46, 47, 48, 49], self‐learning materials (*n* = 12) [12, 21, 22, 24, 25, 27, 28, 34, 36, 41, 46, 49], and then translated self‐learning materials (*n* = 3) [12, 41, 48]. Additionally, there was some variation between the use of community health workers and training community members to perform the education interventions. Further, content covered within these educational interventions varied within studies. However, central themes were consistent: the importance of mammography, what mammography involves, the risk factors of breast cancer and symptoms of breast cancer.

Of the studies (*n* = 5) [41, 42, 43, 44, 45] that used education with culturally adapted content, few studies (2/5) [44, 45] saw improvement in mammogram utilisation. This lacklustre improvement could be due to education being an ineffective behavioural change strategy even with culturally adapted content, or it could be that the individual delivery of this material varies between each study and participant group. The exemplar of the included studies is the Goel et al. study [44], which saw a 22.1% increase in mammogram referral requests, a 20% increase in mammogram completion, a 0.5 out of 10 increase in breast cancer knowledge scores and a 3.7 patient activation score increase. This study used a culturally appropriate video to educate women on the importance of mammography prior to an appointment with their physician. Of the other studies, few studies produced significant changes in mammogram utilisation but had only moderate improvements in breast cancer knowledge [41, 42].

### 3.4 Reminder Systems

Reminder systems took the form of reminder letters, text messages, or phone calls; reminding the patient of their need for a mammogram or upcoming appointment. Most were used in conjunction with other intervention strategies (*n* = 9), while [12, 16, 18, 29, 32, 35, 36, 37, 39] others were reminder systems alone (*n* = 3) [50, 51, 52]. This approach was commonly employed in the primary care setting. When comparing different reminder systems, such as that done in the Fortuna et al. study [51], the most effective mode was the use of two reminder systems together: the use of automated reminder voice messages with in‐person prompts during a primary care visit (28.2% mammography completion). This mode was compared to reminder letters alone (17.8% completion), automated messages with reminder letters (22.8% completion) and reminder letters with personal reminder phone calls (27.5% completion). Similar physical reminder strategies were employed in the Wang et al. study [52], which saw 19.9% of women given the reminder complete mammograms at walk‐in mammogram clinics.

Culturally tailored reminder systems, for example translated reminder letters and reminder calls in preferred language [48, 50], engagement of bilingual community health workers [27, 35] increased the effectiveness of this type of intervention. A review [27] of text messaging reminders had moderate increases in screening rates, particularly in resource‐poor and non‐English‐speaking settings.

### 3.5 Counselling

Counselling approaches in the form of telephone or one‐to‐one counselling were always delivered in combination with other approaches, in particular, education. Telephone counselling was offered in 2 of the studies, with one [49] being particularly effective in Chinese American women who were elderly (65+) and had recently migrated. The study by Wu and Lin saw slight increases among the intervention group (40% mammography completion after 4 months vs. 33% in control group). A longer‐term study, with once a month telephone counselling for 6 months following an education session, showed increases in mammography knowledge (20.2% increase in breast health awareness, 27.8% increase in awareness of mammography) and utilisation (23.6% pre‐intervention intention to complete mammography, 35.4% post‐intervention) [12, 46]. Individual counselling was utilised in one study [45] which combined education sessions with follow‐up phone counselling focused on helping participants overcome barriers to mammography services and was effective in increasing mammography utilisation (50% completion after intervention).

### 3.6 Multi‐Media Interventions

Two review articles explored the impact of multimedia interventions such as messages through phone applications or computer programs. A review of tailored digital interventions had promising results with increases in mammography (up to 75% in intervention vs. 30% in control) when tailored multimedia interventions were coupled with some sort of patient navigation; however, digital literacy and usability are important to consider [25]. Another review [53] found that whilst social media campaigns had the potential for large reach, evidence suggests reach and engagement of CALD populations are much lower in such initiatives. The review also highlighted the challenges with evaluating such campaigns in terms of actual behavior change, with engagement and reach being more common (and easier to measure) outcomes of social media campaigns.

### 3.7 Culturally Appropriate and Tailored Interventions

A cross‐cutting theme across a large portion of the included literature was the incorporation of culturally appropriate, aware, relevant or tailored approaches in some form [12, 16, 17, 19, 20, 21, 22, 23, 24, 25, 26, 27, 28, 29, 31, 33, 34, 35, 36, 37, 38, 40, 41, 42, 43, 44, 45, 46, 47, 48, 49, 50]. This was most commonly through the provision of translated materials or education in language (*n* = 19) [12, 16, 17, 22, 24, 29, 31, 34, 35, 36, 38, 39, 41, 42, 43, 45, 46, 47, 49]. Studies also used translated reminder material [48, 50] to educate courses being offered in specific languages [23, 45, 47] with culturally appropriate content [41]. Multiple review articles referenced the importance and increased efficacy of culturally appropriate and tailored interventions, whether that was multi‐component or singular focus interventions [19, 20, 21, 22, 23, 24, 25, 26, 27, 28, 48, 52].

Another very common approach that considered culture was the engagement of bilingual/bicultural/community workers, educators, or navigators (*n* = 23) [16, 20, 21, 22, 23, 25, 26, 28, 29, 31, 33, 34, 35, 36, 37, 40, 43, 46, 47, 48]. The included studies and review articles linked the efficacy of interventions to employing local workers who lived in the community or were from the same cultural or ethnic group due to cultural appropriateness and acceptability [16, 18, 23, 24, 26, 29, 31, 33, 34, 35, 36, 37, 39, 40, 46, 47, 48, 49]. One review noted that community health worker and patient navigation interventions, in particular, provide an approach that can serve the unique needs of diverse and underserved communities [26].

Culturally relevant material was highlighted in several studies. This included the importance of culturally relevant and familiar graphics or terms for women [12, 19], aligning with cultural constructs including beliefs and practices [12, 19].

Culture was also considered as an opportunity to reach these populations, with recruitment occurring through cultural settings [12, 16, 17, 20, 24, 31, 35, 36, 38, 43, 45, 46] (*n* = 12) such as community organizations and cultural centres [12, 36, 49], faith‐based organizations [12, 16, 20, 24, 34, 35] and resettlement services [17].

Most cultural tailoring focused on the patients. One review paper also mentioned the importance of cultural competence of health care providers [22]. This strategy helped to overcome language and cultural barriers to screening participation. A study within the review described a 2‐h cultural awareness training program for general practice staff, which resulted in a significant increase (19% vs. 5% in control) in mammogram screening attendance in Indian women.

### 3.8 Other

There were 2 studies that did not fit within the intervention categories mentioned above. This included one study that evaluated a model of care for Veteran women, assigning them a ‘designated women's health provider’, which increased mammography use when compared to usual care [54]. Another systematic review [55] looked at the scope and impact of mobile mammography in medically underserved women. This review found that mobile mammography had higher proportions of racial/ethnic minority users and were from lower income households however it was unclear whether this had a sustained impact for repeat mammography use [55].

## 4 Discussion

Access to breast screening (mammography) significantly reduced breast cancer mortality; [4] however, it is well established in the literature that CALD populations experience many barriers to screening, and this has resulted in lower attendance in this group [1, 9, 10]. This review sought to explore available literature on evidence‐based interventions to improve engagement of CALD populations in breast screening in high‐income countries. This review provided 7 core intervention types that have proved to be effective at increasing screening participation of CALD populations: multi‐component, navigation, education, reminder systems, counselling, multi‐media and culturally tailored interventions. The review found the most important consideration of such initiatives is the consideration of culture in terms of cultural appropriateness and tailoring of interventions. This aligns with the broader literature on addressing health inequities, particularly racial/ethnic disparities [56], of which achieving cultural appropriateness in the delivery of health services [57] and health promotion programs [58, 59] is essential.

The review also found that multicomponent interventions were the most commonly utilised and effective in increasing mammography utilisation through screening. This was further evidenced by a number of review studies finding limited efficacy of singular approaches (e.g. reminder phone calls or education alone) when compared with multicomponent interventions [20, 21, 22, 24, 28]. While it is difficult to evaluate each individual component used in the included reviews and intervention studies, there appears to be consensus that studies that used patient navigators, bilingual, and bicultural staff to deliver the intervention have the highest levels of mammography utilisation.

While culturally appropriate materials were present within many of the included multicomponent studies, there were varied levels of improvement in mammogram utilisation [12, 16, 34, 35, 37, 38, 41, 42, 43, 44, 45, 47, 49]. That improvement is also seen when comparing the culturally appropriate education studies [31, 41, 42, 43] and other studies utilising navigation and reminder systems [18, 29, 39, 40]. While the evidence of culturally appropriate materials is varied, included systematic reviews emphasise the importance of this practice to engage CALD populations, and as such, should be considered for strategies seeking to engage CALD populations [19, 20, 21, 23, 24, 25, 28].

There were a number of limitations of this scoping review. The review was limited to peer reviewed articles, and therefore may have missed potentially relevant information in grey literature articles, books, and theses. However, our focus on evaluation assisted in navigating the breadth of the literature. As per scoping review methods [60], presenting an overview of the breadth of studies meant that the included literature was not assessed on quality in terms of bias, validity or generalisability; further, a protocol for this review was not registered.

Another limitation is that most papers included used multiple intervention strategies within individual intervention arms. The use of multiple strategies makes evaluating the impact of individual strategies on mammogram utilisation difficult. Instead, more emphasis is placed on studies that did use intervention arms where one strategy is solely employed, as it demonstrates its individual effectiveness.

Another limitation is the inclusion of predominantly studies from the USA as the differences in healthcare systems make comparing interventions difficult. Given that the US has a predominantly private system, where there are out‐of‐pocket expenses associated with mammograms, it is difficult to separate monetary factors from employed interventions. This is opposed to the Australian system where mammogram services are free for women (who meet the age criteria). Existing literature indicates that the most effective type of intervention to improve screening is providing access to free mammography services [61]. While the search strategy excluded interventions that utilized mammography cost as a primary part of their intervention strategy, it is difficult to compare these private systems to other healthcare systems.

Finally, it should be highlighted that although mammogram utilisation is viewed as an individual behaviour, that is, patients making the decision to participate in screening and much of the current literature [10] and literature in this review target behaviour change, systemic responses are also critical in reaching vulnerable populations. Focusing on individualistic, deficit perspective interventions fails to acknowledge the systemic issues that contribute to health equity. Systemic issues such as addressing barriers to accessing screening and any required follow‐up (affordability, accessibility, availability and appropriate) [62] should be a key consideration of any intervention to improve engagement in screening. This is particularly important to consider when achieving cultural competency in healthcare requires a multi‐level, ecological approach at all levels [63], including addressing the structural challenges that vulnerable populations experience [64].

## 5 Conclusion & Implications

It is well established that CALD populations experience numerous barriers and thus have poorer participation in cancer screening [9, 11, 12]. This scoping review demonstrates the need for further research into the implementation of effective approaches to better engage CALD populations in breast cancer screening. The paper has provided an overview of the core approaches in the international literature that have been effective in increasing mammography utilisation of CALD populations. The literature highlights that the most important consideration is for approaches to be culturally appropriate and tailored. Effective approaches to improve engagement of CALD populations in mammograph utilisation typically included a multicomponent approach, that is, delivering a comprehensive, multi‐pronged approach to improve screening in this population. These findings can be used by policy makers and specific services to inform the development of strategies to better serve their CALD communities and improve their participation in breast cancer screening.

## Author Contributions

**Karla Jaques:** conceptualization (equal), data curation (equal), formal analysis (lead), investigation (equal), methodology (equal), project administration (lead), visualization (equal), writing – original draft (lead), writing – review and editing (lead). **Thomas Baker:** data curation (equal), formal analysis (equal), investigation (equal), visualization (equal), writing – original draft (equal), writing – review and editing (equal). **Deepak Maharaj:** conceptualization (supporting), methodology (supporting), supervision (supporting), validation (equal), writing – review and editing (supporting). **Mohammed Fazli:** conceptualization (supporting), methodology (supporting), writing – review and editing (supporting). **Mandy Williams:** writing – review and editing (supporting). **Patrick Harris:** conceptualization (equal), funding acquisition (lead), methodology (equal), supervision (lead), validation (supporting), writing – original draft (equal), writing – review and editing (equal).

## Conflicts of Interest

The authors declare no conflicts of interest.

## Data Availability

The authors have nothing to report.

## Appendix

**TABLE A1:** Characteristics of Included Studies.

Author and year	Country	Article type	Setting	Target population & sample N	Approach	Intervention type^a^	Relevant outcome measure (s)	Results (screening focused)
Bean‐Mayberry et al. (2015)	USA	Intervention	Primary care	Female veterans (*n* = 37,128, aged 21–69)	•Model of care (Designated Women's Health Provider DWHP)	Model Of Care	Mammography rates	3% increase Odds ratio: 1.24 for DWHP cohort vs. usual care
Beauchamp et al. (2020)	Australia	Intervention (RCT)	National screening program (Breast‐Screen)	Women aged 50–75 years who were due or missed their 2‐yearly screening mammogram. (*n* = 1032 for arm 1, *n* = 195 for arm 2)	Translated reminder letter Written reminder Non‐English Phone call reminder	Reminder Systems, Culturally Tailored	Screening appointment booked within 14‐days	Arm 1: Reminder letters in Arabic or Italian 37.4% Control Arm 1: Reminder letters in English 38.9% Arm 2: Reminder phone calls in Arabic or Italian 64.2% Control Arm 2: No phone call 6%
Borrayo et al. (2017)	USA	Intervention	Community	Spanish‐speaking Latinas (*n* = 141)	Non‐English video education Bilingual education—Non‐English written education Culturally appropriate	Education, Culturally Appropriate	Breast cancer knowledge, self‐efficacy, behavioural norms and behavioural intentions as it relates to mammography	Narrative and nonnarrative interventions were equally effective for increasing Latinas' bc knowledge and perceived behavioural norms Narrative intervention was significantly higher mammography self‐efficacy (confidence)
Brown et al. (2019)	USA	Intervention	State‐based screening program	Low income, uninsured and underinsured people of Louisiana (*N* = 4007)	Patient navigation One‐on‐one education Bilingual education	Patient Navigation, Education, Culturally Tailored	Proportions of individuals attending mammography appointments through the navigation system and free cost program	The program met it targets for Black women (47%) and exceeded its targets for Latina women (18%). Improving access for these populations using navigation
Castaneda et al. (2016)	USA	Intervention	Primary care	Latinx, Spanish speaking individuals who attended the clinic (*n* = 107)	Culturally tailored Group education Non‐English group education	Education, Culturally Tailored	Breast cancer knowledge & Screening intentions	Knowledge: Pre: 2.64% Post: 3.02% Intention: Pre: 95.2% Post: 97.6%
Chan & Winnie (2015)	Various, USA & Canada	Review Article (Systematic review)	Community or Healthcare	RCTs included = 10 Ethnic minority	Health Promotion Programmes (education, culturally tailored materials)	Education, Culturally Tailored	Screening intention or uptake, screening knowledge, health beliefs of bc and screening	Key Characteristics for Effective Programmes: All 10 used linguistically appropriate methods Culturally relevant strategies Instruction in community settings Content with key messages about both forms of cancer and screening Multiple intervention strategies
Cullerton et al. (2016)	Australia	Intervention	Community	CALD (*n* = 159 total, *n* = 69 attending the breast cancer education sessions)	Culturally appropriate Non‐English group Education Sessions performed by trained multicultural health workers	Education, Culturally Appropriate	Knowledge, attitudes, intention to screen as well as mammography rates	Knowledge and attitudes improved overall. Mammography rates: No change (46%)
Falk et al. (2022)	USA	Intervention	Community	Women aged 40–73 (*n* = 4942)	Group Education Patient navigation Bilingual education	Education, Patient Navigation, Culturally Tailored, Culturally Appropriate	Mammography rates	Arm 1: Patient navigation 74.3% Arm 2: Patient navigation with education 62.9% Arm 3: Education alone 28.4%
Fortuna et al. (2013)	USA	Intervention (RCT)	Primary care	Women 40–74 (*N* = 624)	Automated telephone call reminder Written reminder prompt (in‐person) Letter reminder Personal phone call reminder	Reminder Systems, Culturally Tailored	Mammography rates	Arm 1: Reminder letter only (control) 17.8% Arm 2: Automated call with a reminder letter 22.8% Arm 3: Automated phone call with reminder letter and written prompt 28.2% Arm 4: Reminder letter with personal phone call 27.5%
Goel et al. (2016)	USA	Intervention	Primary care	Convenience sample of women from the primary care patients (*n* = 97)	Video Education Culturally appropriate	Education, Culturally Appropriate	Mammography referral request rates, mammography completion, knowledge, and patient activation	Arm 1: Video before appointment (culturally appropriate) Arm 2: No video (regular care i.e. control) Mammography referral request rates: Arm 1: 36.7% Arm 2: 14.6 Mammography Completion: Arm 1: 33 Arm 2: 13% Knowledge: Arm 1: 8.4/10 Arm 2:7.9/10 (not significant)
Gondek et al. (2015)	USA	Intervention	Community	Immigrant and refugee females (*n* = 348)	Culturally appropriate Group Education sessions Patient navigation Bilingual education	Education, Navigation, Culturally Appropriate	Cancer knowledge, cancer awareness and mammogram completion	Knowledge improvement in all items Mammography completion: Before: 0% After: 35%
Han et al. (2017)	USA	Intervention (RCT)	Community	Korean American Women (*n* = 560)	One‐on‐one educational phone calls Group Education sessions Educational brochures Patient navigation Bilingual education Self‐learning education materials	Education, Counselling, Patient Navigation, Culturally Tailored	Health literacy, cancer knowledge, perceptions about cancer screening, Mammogram completion	Mammography Completion: Arm 1: Individually tailored education brochures tailored to the individuals own risk factors, one‐on‐one counselling, education, self‐learning materials: 56.1% Arm 2: Educational brochures (control) 10%
Henderson et al. (2020)	USA	Intervention	Community	Women within the 5 Federally qualified Health Centres (*n* = 546)	Patient Navigation Bilingual navigators who also provide education Reminder Letters Education sessions & Education brochures Outreach activities	Patient Navigation, Education, Reminder Systems, Outreach, Culturally Tailored	Mammogram completions	Before: 103 in 12‐month period After: 567 in 12‐month period
Hou et al. (2018)	USA	Review Article (Systematic review)	Community	Racial/Ethnic Minority Groups (African American, Latino/Hispanic, Korean Americans, Samoan Americans) 20 Studies	Cancer education delivered in Faith‐based setting Client reminders Group Education One‐on‐one education	Education, Reminder Systems, Education, Culturally Tailored & Appropriate	Knowledge, screening utilisation, beliefs, accessibility, perceived barriers to screening, vaccination intent	Lessons for effectiveness: Cancer education effective in increasing knowledge and beliefs Cultural tailoring improves screening rates
Kim et al. (2022)	Korea	Intervention	Community	Chinese married immigrant women in Korea (*n* = 183)	Self‐learning (DVDs & brochure) One‐on‐one education Bilingual education (bicultural community health workers) Telephone counselling	Education, Counselling, Reminder Systems, Culturally Tailored	Mammography and pap test knowledge and utilisation	Arm 1: one‐on‐one education, phone call counselling, bilingual. Before: 23% After: 31.1% Arm 2: Educational materials (brochures) Control Before: 16.4% After: 0%
Kwok & Lim (2016)	Australia	Intervention	Community	Chinese‐Australian Women (*n* = 288)	Culturally tailored Self‐learning education materials Group education session Non‐English group education & self‐learning materials Calendar reminders	Education, Reminder Systems, Culturally Tailored	Awareness of screening practices, screening intention and knowledge of breast and cervical cancer	Mammogram intention: Before: 23.6% After: 35.4%
Lee‐Lin et al. (2013)	USA	Intervention	Community	Chinese American Immigrant Women (*n* = 44)	Culturally appropriate (‘Culturally responsible) Non‐English Group education	Education, Counselling, Culturally Appropriate	Mammography use, readiness to change, knowledge, perceptions and barriers	50% mammography completion after program (*n* = 21)
Liu et al. (2023)	USA	Review Article (Systematic review)	Community	Rural and farmworker communities	Culturally appropriate One‐on‐one education Group education Self‐learning education Reminder phone calls Educational videos	Education, Reminder Systems, Culturally Appropriate	Screening uptake	Most effective interventions for this population: Use of lay health workers to provide education Culturally relevant interventions For farmworkers: direct involvement of cultural leaders
Lu et al. (2012)	Various (USA, Taiwan, Thailand, UK, Canada, Singapore, Australia, New Zealand, Hong Kong, India and Malaysia)	Review Article (Systematic review)	Multiple (community, workplace, media)	Asian Women (37 studies)	Patient navigation Culturally appropriate Group education Self‐learning materials Reminder letters One‐on‐one education Bilingual education	Patient Navigation, Education, Reminder Systems, Culturally Appropriate, Culturally Tailored	Mammogram usage/attendance	Most effective interventions: Combination of multiple strategies Interventions improved with support to attend (e.g. assistance scheduling
Luque et al. (2019)	USA	Review Article (Systematic review)	Community	Hispanic Women (5 studies)	One‐on‐one education Group education Culturally appropriate Screening education interventions	Education, Culturally Appropriate	Increased mammography use	Low to moderate intervention affect from: Promotora approach (community health worker) Motivational, interpersonal education sessions (one‐on‐one or group)
Mojica et al. (2015)	USA	Intervention	Community	Hispanic Women aged 18–75 (*n* = 691)	Group Education Bilingual education Bilingual patient navigation Phone reminder	Education, Patient Navigation, Reminder Systems, Culturally Tailored	Increased cancer screening, knowledge of screening guidelines and benefits	After intervention 25% of the sample had completed a mammogram with an additional 14% on the waitlist for one
Molokwu et al. (2023)	USA	Intervention	Community	Hispanic Women 50–75, uninsured or underinsured and overdue for screening (*n* = 600, 300 control, 300 intervention)	Culturally appropriate Bilingual education Bilingual navigation Education Reminder calls	Education, Patient Navigation, Reminder Systems, Culturally Tailored, Culturally Appropriate	Mammogram screening 4 months post‐intervention	Arm 1: Culturally tailored, bilingual education, navigation, free mammogram: 97% Arm 2: Usual care (control) 4.4%
Nair et al. (2022)	USA	Intervention	Community	Uninsured women enrolled into the NBCCEDP‐funded program of North Texas aged 40–64 (*n* = 19,292)	Telephone navigation Letter reminders	Patient Navigation, Reminder Systems	Mammogram adherence (second mammogram and beyond)	Baseline: Non‐adherence: 72.1% Adherent: 27.9 Longitudinal adherence: 81.1%
Nguyen‐Truong et al. (2017)	USA	Intervention	Community	Vietnamese American women, aged 50 years or older (*n* = 40)	Group education One‐on‐one education (described at counselling) Bilingual education Bilingual Patient navigation Culturally appropriate	Education, Counselling, Patient Navigation, Culturally Appropriate, Culturally Tailored	Stage of readiness change improvement and mammogram completion (12 weeks after intervention)	Mammogram completion rate of 75% at the end of the study
Offman et al. (2014)	UK	Intervention	Primary care	Women aged 50–70 (*n* = 10,928)	Telephone reminder calls Reminder letters Culturally appropriate Patient navigation	Reminder Systems, Patient Navigation, Culturally Appropriate	Mammogram uptake	Increased mammography of 15%
Okasako‐Schmucker et al. (2023)	Various (USA, Australia, Belgium, Canada, Hong Kong, China, and United Kingdom)	Review Article (Systematic review)	Various	Various (39 papers)	Community Health Worker (CHW) One‐on‐one education Self‐learning education Culturally appropriate Bilingual Patient navigation Group education	Model of Care, Education, Patient Navigation, Culturally Appropriate, Culturally Tailored	Screening uptake	Using a CHW saw 11.5% increase in screening CHW alone 9.2% (4.7%–22.8%) CHW added 11.0% (2.3%–13.5%) CHW in a team 13.7% (9.1%–29.7%)
Percac‐Lima et al. (2012)	USA	Intervention	Community	Refugee Serbo‐Croation women who were overdue for or had never had a mammogram (*n* = 95)	Bilingual Patient navigation and education Culturally appropriate (‘culturally tailored navigation program’) Reminder telephone calls Phone education	Patient Navigation, Reminder Systems, Education, Counselling, Culturally Appropriate, Culturally Tailored	Mammography screening completion	Before: 44% After: 67%
Percac‐Lima et al. (2013)	USA	Intervention	Community	Refugees 40–74 (*n* = 188)	Bilingual Patient navigation and education Patient navigation Group education Culturally appropriate Over the phone counselling (and education)	Patient Navigation, Reminder Systems, Education, Counselling, Culturally Appropriate, Culturally Tailored	Mammography rates	Refugee group: Before: 64.1% After: 81.2% English‐speaking group: Before: 76.5% After: 80.0%
Percac‐Lima et al. (2016)	USA	Intervention (RCT)	Community	Women aged 50–74	Bilingual Patient navigation and education Over the phone counselling (and education) Written reminder letters Reminder phone calls Patient navigation	Patient Navigation, Education, Counselling, Culturally Tailored	Mammography screening completion	Arm 1: bilingual navigation, counselling, reminder letters, reminder phone calls: 23.4% Arm 2: reminder letters, reminder phone calls (control): 16.6%
Plackett et al. (2020)	Various (USA, UK, Canada, Europe, South Korea, Taiwan, South America, Saudi Arabia, Nigeria)	Review Article (Systematic review)	Community	Various national, untargeted general public and targeted (23 publications)	Social media campaigns	Social Media	Level of engagement (exposure, reach) behaviour change (intention to screen and screening)	Lessons learnt: Observational approach commonly used: low to medium engagement with campaign Evidence of less reach and engagement from ethnic minority groups Providing actionable health messaging influences behaviour change more than fundraising messages Recommends use of evaluation frameworks‐ measuring impact in terms of behaviours change is difficult
Racine et al. (2023)	Various (USA, Israel, Turkey & Jordan)	Review Article (Systematic review)	Various	Muslim refugee and migrant women (14 studies)	Patient navigation Culturally appropriate Bilingual education One‐on‐one education Group education Self‐learning materials Navigation services in a faith based setting	Patient Navigation, Education, Culturally Appropriate	Breast Self Examination, clinical breast examination & mammogram screening rates	Effectiveness: Multi‐disciplinary approach is needed Stand alone interventions are less effective Cultural and religious barriers must be addressed Education and patient navigation are effective when used together Community based cultural and faith based interventions are most effective
Richardson‐Parry et al. (2023)	Various (USA, Denmark and Hong Kong)	Review Article (Systematic review)	Various (Community & Primary Care)	Multiple: low socioeconomic status or specific cultural or ethnic/racial groups (17 studies)	Self‐learning materials (DVD) Group education Culturally appropriate Patient navigation Multimedia	Education, Patient Navigation, Multimedia, Culturally Appropriate	Screening completion	Interventions that use individual and cultural tailoring of cancer screening educational material show promise however, digital literacy and useability are important to consider
Richman et al. (2020)	USA	Intervention	Community	Uninsured and underinsured Black and Latina women (*n* = 735)	Culturally appropriate Group education Patient navigation Bilingual patient navigation	Education, Patient Navigation, Culturally Appropriate	Clinical breast exams and mammograms	Patients who received navigation and were recommended for mammogram: 71% received Of the group who had never received mammogram, 68% received one
Rodriguez‐Torres et al. (2019)	USA	Intervention	Primary care	Refugee women	Bilingual patient navigation (including education) Culturally appropriate	Patient Navigation, Education, Culturally Appropriate	Screening completion	When program ended, screening proportions were significantly higher among the refugee women (90.5% vs. 81.9%) 5 years post: refugee women's screening was comparable with English‐speaking women (76.5%, 80.5%)
Roland et al. (2017)	USA	Review Article (Systematic review)	Community & Primary Care	Medically underserved communities (24 articles)	Patient navigation Community Health Worker	Patient Navigation, Model of Care	Completion of cancer screening test, timely follow up (diagnosis) and referral for screening by CHW/PN	Effectiveness: Most studies found significant positive outcomes of CHW/PN interventions CHW/PN improve completion and timeliness of screening and diagnosis CHW/PN interventions can be applied in both clinical (primary care) and community setting
Schoueri‐Mychasiw et al. (2013)	Various (UK, Canada, Australia)	Review Article (Systematic review)	Multiple (primary care, community, mass media)	Immigrant and Minority Women (8 studies)	Reminder letters Reminder calls Multimedia (mass media messages) Patient navigation (language support) Group education sessions One‐on‐one education Multiple (Screening reminders, translated materials, Addressing barriers e.g. transportation, GP referral)	Reminder Systems, Multimedia, Patient Navigation, Education	Screening attendance	Focused on overcoming barriers including language and transportation barriers and increasing cues to screening 6/8 studies reported increases in screening between 5% and 70% Most important factor: addressing and accommodating for differences in cultural beliefs
Torres et al. (2019)	USA	Intervention	Community	Uninsured/underinsured women in rural setting (*n* = 735)	Culturally appropriate Bilingual education Patient navigation (telephone) Education	Education, Patient Navigation, Culturally Appropriate	Education received, breast health assessment, mammogram utilisation	After intervention: 72% received mammogram screening
Uy et al. (2017)	Various (Spain, England, USA, Malaysia, Israel)	Review Article (Systematic review)	Community & Primary Care	Clinical trials for text messaging for 4 cancers: breast, cervical, colorectal and lung (9 studies)	Text message reminder Self‐learning (Brochure—used as a control)	Reminder Systems, Education, Culturally Tailored	Screening rates	All text messaging interventions increased screening rates ranging between 4.5% and 15% (absolute) and 20%–63% (relative) The largest increase: individualised and culturally tailored for Korean American Women
Vang et al. (2018)	USA	Review Article (Systematic review)	Community	Medically underserved women (10 studies)	Mobile mammography service	Mobile Mammography	Repeat visits, screening adherence and recency of screening, screening outcomes & perceived risk	Efficacy: Mobile mammography services have good reach of underserved women (low income, ethnic minority and uninsured) Low adherence in longer term
Wang et al. (2020)	USA	Intervention	Community & Primary Care	Women who attended mammography at a community‐based breast imaging centre (*n* = 3688)	In‐person reminders (for physicians) Physician (primary care) reminders for mammography	Reminder Systems	Mammography use, repeat mammograms	Card for walk‐in screening were used by 1/5 women. Cards were more likely to be used by ethnic minorities, limited English proficiency and Medicaid insured.
Wu & Lin (2015)	USA	Intervention (RCT)	Community	Chinese American Women (*n* = 193)	One‐on‐one education (phone) Telephone counselling Culturally tailored Group education Bilingual education Self‐learning (brochure)	Education, Counselling, Culturally Tailored, Culturally Appropriate	Mammography completion, screening behaviours, knowledge	Arm 1: tailored counselling phone call, culturally appropriate, bilingual: 40% Arm 2: Self‐learning brochures on breast cancer and mammograms (control): 33%

^a^ It should be noted the difference between ‘culturally appropriate’ and ‘culturally tailored’ interventions: Cultural appropriateness is an intervention that is effective for the population it serves, for example, using lay/community health workers or education materials that have been developed by the target community whereas, cultural tailoring are strategies to make a program more appropriate, for example, translation of education materials or use of interpreter [1, 2].
